# Retraction Note: Inhibition of acetylation of histones 3 and 4 attenuates aortic valve calcification

**DOI:** 10.1038/s12276-026-01681-8

**Published:** 2026-02-20

**Authors:** Jia Gu, Yan Lu, Menqing Deng, Ming Qiu, Yunfan Tian, Yue Ji, Pengyu Zong, Yongfeng Shao, Rui Zheng, Bin Zhou, Xiangqing Kong, Wei Sun

**Affiliations:** 1 Department of Cardiology, The First Affiliated Hospital of Nanjing Medical University, 300 Guangzhou Road, 210029 Nanjing, PR China; 2 Department of Cardiothoracic Surgery, The First Affiliated Hospital of Nanjing Medical University, 300 Guangzhou Road, 210029 Nanjing, PR China; 3 Departments of Genetics, Pediatrics, and Medicine (Cardiology), The Wilf Cardiovascular Research Institute, The Institute for Aging Research, Albert Einstein College of Medicine, Bronx, NY 10461 USA

Retraction Note to: *Experimental & Molecular Medicine* 10.1038/s12276-019-0272-9, published online 10 July 2019

The Editor-in-Chief has retracted this article. Following publication, a concern was raised regarding Fig. 1B. A correction was submitted and the article was updated but further investigation has identified that the original images supplied appear not to be from the time of the original experiment. The animal ethics approval documents were also found to be insufficient. The Editor-in-Chief has therefore lost confidence in the data and conclusions of this article.

The authors have not responded to correspondence from the publisher regarding this retraction.

